# Effects of bacteriocin-producing *Lactiplantibacillus plantarum* on bacterial community and fermentation profile of whole-plant corn silage and its in vitro ruminal fermentation, microbiota, and CH_4_ emissions

**DOI:** 10.1186/s40104-024-01065-w

**Published:** 2024-08-07

**Authors:** Ziqian Li, Samaila Usman, Jiayao Zhang, Yixin Zhang, Rina Su, Hu Chen, Qiang Li, Mengya Jia, Tunde Adegoke Amole, Xusheng Guo

**Affiliations:** 1 School of Life Sciences, Lanzhou University, Lanzhou, 730000 P.R. China; 2 Probiotics and Life Health Research Institute, Lanzhou University, Lanzhou, 730000 P.R. China; 3 International Livestock Research Institute (ILRI), IITA Campus PMB, Oyo Road, Ibadan, 5320 Nigeria

**Keywords:** Bacteriocin-producing *Lactiplantibacillus plantarum*, Fermentation, Methane emissions, Microbiota, Rumen, Whole-plant corn silage

## Abstract

**Background:**

Silage is widely used to formulate dairy cattle rations, and the utilization of antibiotics and methane emissions are 2 major problems for a sustainable and environmentally beneficial ruminant production systems. Bacteriocin has received considerable attention because of its potential as an alternative to antibiotics in animal husbandry. However, the impact of bacteriocin-producing lactic acid bacteria on the microbiological conversion process of whole-plant corn silage and rumen fermentation remains limited. The purpose of this study was to assess the effect of 2 class IIa bacteriocin-producing strains *Lactiplantibacillus plantarum* ATCC14917 and CICC24194 on bacterial community composition and ensiling profiles of whole-plant corn silage and its in vitro rumen fermentation, microbiota, and CH_4_ emissions.

**Results:**

Both bacteriocin-producing strains increased the lactic acid concentration in silage fermented for 7 d, whereas the lowest lactic acid was observed in the ATCC14917 inoculated silage fermented for 90 d (*P* < 0.05). The highest DM content was observed in the CICC24194 treatment (*P* < 0.05), and the silages treated with both strains had the lowest DM loss (*P* < 0.05). Bacteriocin-producing strains promoted the growth of *Levilactobacillus brevis* on d 60 of ensiling. In addition, treatment with bacteriocin-producing strains increased the in vitro DM digestibility (*P* < 0.05) and decreased the CH_4_ production (*P* < 0.05). The results of random forest and clustering analyses at the genus level showed that ATCC14917 increased the relative abundance of the influential variable *Bacillus* compared to that in the control group, whereas CICC24194 decreased the relative abundance of the influential variable *Ruminococcaceae* UCG-005. The CICC24194 treatment had the lowest total bacterial, fungal, protozoan, and methanogen populations (*P* < 0.05).

**Conclusions:**

Both class IIa bacteriocin-producing *L. plantarum* strains improved the fermentation quality of whole-plant corn silage by regulating the bacterial community composition during ensiling, with CICC24194 being the most effective. Both bacteriocin-producing strains mitigated CH_4_ production and improved digestibility by modulating the interactions among rumen bacteria, protozoa, methanogens, and the composition of fibrolytic bacteria.

**Supplementary Information:**

The online version contains supplementary material available at 10.1186/s40104-024-01065-w.

## Introduction

Worldwide cultivation of whole-plant corn for silage is due to its high nutritional value, palatability, economical price, and stable growth in a variety of environments [1, 2]. The amount of corn grown for whole-plant silage has significantly increased over the last few decades, second only to forage for grass. Producing whole-plant corn silage (WPCS) is widely practiced due to its high nutritional value, palatability, economical price, and growth. However, any slight penetration of oxygen during silage compaction can allow the multiplication of yeasts and *Clostridium*, resulting in severe dry matter (DM) loss [3, 4]. Enteric methane (CH_4_) is one of the main sources of greenhouse gases produced by the loss of digestive energy in ruminants [5, 6]. Ruminants contribute to approximately 16% of the global greenhouse gas emissions [7]. Hence, reducing DM loss and CH_4_ emissions from WPCS are two major challenges in the development of silage processing and animal husbandry.

Probiotic silage additives from lactic acid bacteria (LAB) have been developed to improve the silage fermentation quality and animal productivity [8]. Bacteriocin, a strain-specific antimicrobial peptides released by bacteria, has received considerable attention because of its resistance-free, residue-free, and environmentally beneficial properties as well as its potential as an alternative to antibiotics in animal husbandry [9, 10]. Based on the available information, the application of bacteriocin-producing LAB not only improved alfalfa silage fermentation but also reduced rumen CH_4_ emissions [11–13].

The silage fermentation was mainly dominated by LAB, and the succession of microbial community in raw materials showed a strong correlation with the fermentation quality [14]. Ruminal microorganisms comprise a complex microcosm that plays an important role in digestion. Previous studies have explored the variability in microbial communities during silage and rumen fermentation to reveal their biological processes [15–17]. However, further understanding of the impact of different bacteriocin-producing LABs on the microbiological conversion process of WPCS and rumen fermentation remains limited. Due to the antibacterial effect of bacteriocin-producing LAB, we hypothesized that its application in silage fermentation may suppress the proliferation of spoilage bacteria during fermentation, and regulate the rumen microecology. These actions can preserve silage DM and remission CH_4_ emissions in the rumen. Thus, this study aimed to investigate the effect of treating WPCS with different bacteriocin-producing *Lactiplantibacillus plantarum* strains on the bacterial community and fermentation parameters, as well as in vitro ruminal fermentation characteristics, microbiota, and CH_4_ emissions.

## Materials and methods

### Inoculated strain preparation

The strains used in this study included *L. plantarum* ATCC14917 and CICC24194, class IIa bacteriocin-producing strains purchased from the China Center of Industrial Culture Collection [18, 19]; *L. plantarum* MTD/1 (NCIMB 40027), a commercial silage inoculant (Ecosyl, Volac International Limited, Hertfordshire, UK). Before silage preparation, the strains were activated twice every 8 h in deMan Rogosa Sharpe (MRS) broth at 1% (v/v) and incubated at 37 °C for 18 h after the second activation.

### Whole-plant corn silage preparation

Whole-plant corn (*Zea mays* L. Dajingjiu3876) was reaped with a forage harvester at half milk line from 4 random fields (served as replication for each treatment) in an established farm located in Dingxi (N35°58′, E104°62′), Gansu Province, China. The DM content of fresh whole-plant corn was 277 g/kg of fresh weight (FW), and the crude protein (CP), water-soluble carbohydrates (WSCs), aNDF (neutral detergent fiber with heat-stable α-amylase), acid detergent fiber (ADF), and starch contents were 55.9, 318, 465, 269, and 292 g/kg DM, respectively. Wilted whole-plant corn from each field was chopped into small pieces (approximately 2 cm) and divided into 5 piles for 5 fermentation periods (3, 7, 14, 60, and 90 d). The following treatments were applied to 5 piles from the 4 fields: (1) distilled water (control), (2) MTD/1 strain (MTD/1), (3) ATCC14917 strain (ATCC14917), and (4) CICC24194 strain (CICC24194), applied to 1 × 10^5^ colony-forming units (CFU)/g FW. In addition, approximately 500 g of chopped whole-plant corn from each field was stored as fresh samples at –20 °C for subsequent analysis. Approximately 500 g of silage from each sub-pile was sealed in a vacuum plastic bag (density 0.91 to 0.93 g/cm^3^; vacuum degree 0.1 Mpa).

### Fermentation profile and chemical composition analyses of whole-plant corn silage

During each ensiling period, 20 g of sample (fresh and silage samples) was collected from each mini-silo, homogenized with 180 mL of distilled water, and filtered through 4 layers of gauze. The pH of the filtrate was immediately measured using an acidometer (PB-10; Sartorius, Gottingen, Germany). A portion of the filtrate was acidified to pH 2.0 with H_2_SO_4_ (7.14 mmol/L) and filtered with a 0.22-μm filter for subsequent organic acid (lactic, acetic, propionic, and butyric acid) analysis. The organic acids were determined using high-performance liquid chromatography (HPLC, KC-811 column, Shodex, Shimadzu, Tokyo, Japan; oven temperature: 50 °C; flow rate: 1 mL/min; SPD: 210 nm) following the method of Ke et al. [20]. Another portion of the unacidified filtrate was used to determine the non-protein nitrogen (NPN), ammoniacal nitrogen (NH_3_-N), and WSC concentrations according to the methods of Licitra et al. [21], Broderick and Kang [22], and Murphy [23], respectively. An air-force oven (FED260, Binder, Neckarsulm, Germany) was used to dry the fresh and ensiled samples at 65 °C for 72 h. After determining the DM content, the dried samples were ground with a 1-mm sieve mill to determine the total nitrogen (TN) and CP contents using an automatic Kjeldahl apparatus (NKY6120, FOSS, Hilleroed, Denmark) according to Licitra et al. [21]. aNDF and ADF were determined using a fiber analyzer (Fibertec 8000, FOSS, Hilleroed, Denmark) according to the methods described by Thiex [24].

### In vitro rumen substrate and incubation

Ground samples of whole-plant corn ensiled for 90 d were used for in vitro ruminal fermentation trials. Three rumen-fistulated Hu sheep (Taihu Lake region, Zhejiang Province) with a mean body weight of 60 ± 5 kg were selected as rumen fluid donors. The sheep were fed twice daily (06:00 and 18:00) with a total mixed ration (TMR) pellet containing 58% corn, 19% wheat bran, 18% soybean meal, 1% baking soda, and 4% vitamin and mineral supplements. Fresh rumen fluid was collected before morning feeding. The collected rumen fluid from each donor was filtered through 4 layers of gauze and mixed in equal volumes. The mixed rumen fluid roundly poured into a 1,500 mL thermostatic (39 °C) sterile bottle filled with CO_2_. The rumen sample was promptly taken to the laboratory within 20 min and continuously infused with CO_2_ until use. Preparation of the anaerobic buffer solution and in vitro fermentation were conducted following the description of Li et al. [13]. Each 1 L artificial buffer solution (pH 7.0) contains 237 mL buffer solution (4.0 g/L NH_4_HCO_3_ + 35.0 g/L NaHCO_3_), 237 mL macro element solution (5.7 g/L Na_2_HPO_4_ + 6.2 g/L KH_2_PO_4_), 0.12 mL trace element solution (13.2 g/100 mL CaCl_2_∙2H_2_O + 10.0 g/100 mL MnCl_2_∙2H_2_O + 1.00 g/100 mL CoCl_2_∙6H_2_O + 8.00 g/100 mL FeCl_3_ + 6H_2_O), 1.22 mL resazurin solution (100 mg/100 mL), 50 mL reducing agent solution (285 mg/50 mL Na_2_S∙7H_2_O + 800 mg/50 mL NaOH), and 474 mL distilled water. After preheating the buffer to 39 °C and continuously infusing CO_2_, 20 mL filtrate was mixed with 70 mL buffer in a sterile glass sealed bottles (100 mL). A ground sample (0.5 g) from each mini-silo was prepared in quadruplicate (a total of 64 subsamples, 16 replicates per treatment), weighed into a fiber bag (F57, Ankom Technology, New York, USA) of constant weight, and heat-sealed. Three more bags were used as blanks. All bags were placed in sterile glass bottles. Two bottles of each silage replicate were linked to an automatic microbial fermentation gas production recorder (Boxiang Xingwang Technology Co., Ltd., Beijing, China) to evaluate the total gas production. The other two bottles were linked to gas-collection bags (500 mL) for subsequent CH_4_ production analyses. All bottles were cultured in a thermostatic water bath (280C, Kuanson Biotechnology Co., Ltd., Shanghai, China) for 48 h at 39 °C.

### In vitro ruminal fermentation characteristics and CH_4_ production analysis

After 48 h incubation, the fiber bags were cleaned with warm water 5 times and dried at 105 °C for 3 h to calculate the in vitro DM digestibility (IVDMD). The incubated fluid was treated with 25% H_3_PO_4_ containing an interior label of 2-methyl butyric acid for subsequent volatile fatty acid analysis using gas chromatography (trace 1300, Thermo Fisher Scientific) with an electrical conductivity detector and capillary column (30 m × 0.32 mm × 0.50 µm; Lanzhou Zhongke Kaidi Chemical New Technology Co., Ltd, Lanzhou, China) following the method described by Chen et al. [25]. The ammonia (NH_3_-H) concentration was assayed according to the method described by Broderick and Kang [22]. The collected gas was used to determine the CH_4_ production using gas chromatography with a flame ionization detector and 19095P-QO3 column (30 m × 0.53 mm × 40.00 µm; Agilent Technologies Inc., CA, USA) according to the method of Chen et al. [25].

### Microbiological analysis

Total bacterial DNA in silage and in vitro incubated fluid were extracted. Briefly, silage samples (15 g) were mixed with sterile saline solution (50 mL) and shaken at 25 °C at 120 r/min for 10 min. The shaken liquid was then filtered through 8 layers of gauze. After centrifugation at 4 °C at 10,000 × *g* for 10 min, the precipitate of the silage and in vitro incubated fluid (1 mL) was collected (bacterial cells). Total DNA was extracted from the precipitate, and its concentration and purity were assessed as described by Bai et al. [26].

The 16S rRNA amplification of the silage and in vitro incubated fluid was performed using specific primer—V1–V9 (27F: 5′-AGRGTTYGATYMTGGCTCAG-3′; 1492R: 5′-RGYTACCTTGTTACGACTT-3′) and V3–V4 (338F: 5′-ACTCCTACGGGAGGCAGCA-3′; 806R: 5′-GGACTACHVGGGTWTCTAAT-3′). The amplicon libraries of the silage filtrate and rumen fluid were sequenced using PacBio Sequel II and Illumina Nova 6000 (Magigene Biotechnology Co., Ltd., Guangzhou, China), respectively. The UPAPSE platform and Silva database were used for Operational Taxonomic Units clustering and species annotation of the sequencing data, respectively. The species or genus community structure, beta diversity, fermentation factor correlation analysis, and random forest analysis were performed using the R software.

### Quantitative real-time PCR analysis

The primers used for real-time PCR quantification of total bacteria, fungi, protozoa, and methanogens are listed in Table S1. Standard preparation was carried out by monoclonal sequencing of the PCR-recovered products after retrieval of the target gene. Production of the standard curve involved performing a quantitative PRC reaction by diluting the standard to 8 concentrations. The same primers and conditions were used according to the standard curve, and the samples were analyzed by qPCR in 3 replicates. Standard plasmids were used as positive controls for error correction, and NTC was used as a negative control.

### Statistical analysis

The data on silage fermentation parameters were analyzed using the general linear model of SPSS (version 22.0; IBM Co., Armonk, NY, USA) according to a 5 × 4 factorial experiment, as in the model below:

1 $$Y_{ijk}=\mathrm\mu+{\mathrm\alpha}_i + \beta_{j} +{\left(\alpha\times\beta\right)}_{ij}+e_{ijk,}$$

where *Y*_*ijk*_ represents the dependent variables; *μ* is the overall mean; *α*_*i*_ is the effect of ensiling period *i* (*i* = 3, 7, 14, 60, and 90), *β*_*j*_ is the effect of treatment *j* (*j* = control, MTD/1, ATCC14917, and CICC24194), (*α* × *β*)_*ij*_ is the effect of interaction between ensiling periods and treatments, and *e*_*ijk*_ is the error term. Comparisons among treatments within each ensiling period were made using Tukey’s test when at least one of the contrasts of ensiling period × treatment was significant. Differences were considered as significant at *P* < 0.05.

Data on the chemical composition of silage fermented for 90 d and ruminal fermentation parameters were subjected to one-way analysis of variance. Tukey’s multiple comparison test was used to separate significant means at *P* < 0.05.

## Results

### Fermentation profile and chemical characteristics of whole-plant corn silage inoculated with different LAB strains

**Fig. 1:**
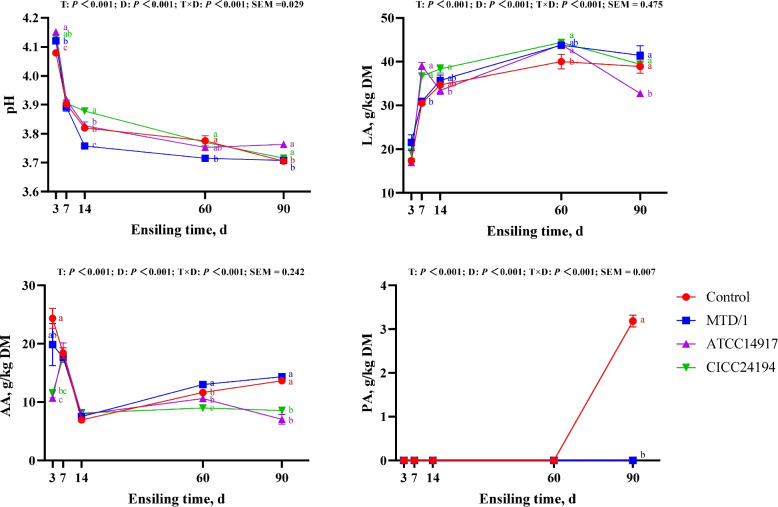
Fermentation parameters of whole-plant corn silage. LA, lactic acid; AA, acetic acid; PA, propionic acid; DM, dry matter. Control, distilled water; MTD/1, MTD/1 treatment; ATCC14917, ATCC14917 treatment; CICC24194, CICC24194 treatment. ^a–c^Means with different superscript letters show differences among treatments at each ensiling period (*P* < 0.05). Red letters, control; bule letters, MTD/1 treatment; purple letters, ATCC14917 treatment; green letters, CICC24194 treatment. SEM, standard error of means. T, the effect of treatments; D, the effect of ensiling periods; T × D, the interaction of treatment and ensiling periods

The fermentation parameters of the WPCS are shown in Fig. 1. There were treatment × DM × ensiling period interactions (*P* < 0.001) for pH, lactic acid, acetic acid, and propionic acid. Both bacteriocin-producing strains increased the lactic acid concentration in silage fermented for 7 d, whereas the lowest lactic acid was observed in the ATCC14917 inoculated silage fermented for 90 d (*P* < 0.05). The butyric acid was not present in any of the ensiling treatments. The propionic acid was detected only in the control group after 90 d of ensiling. The lowest pH was observed in the control group at the initial stage of fermentation (d 3); however, at the late stage of fermentation (90 d), higher pH values were observed in the silage treated by bacteriocin-producing strains compared with control and MTD/1 treatment (*P* < 0.05). The control group had the highest acetic acid concentration on d 3 of ensiling, whereas an increasing acetic acid concentration was observed in the MTD/1 treated silage on d 60 compared with that in the control group (*P* < 0.05). When silage fermentation reached d 90, the MTD/1 and control groups showed the highest acetic acid concentrations (*P* < 0.05).

The chemical composition of WPCS fermented for 90 d is reported in Table 1. All 3 inoculants increased the DM content and decreased the DM loss of WPCS (*P* < 0.05). The highest DM content was observed in the CICC24194 treatment (*P* < 0.05), and both bacteriocin-producing strain-treated silages had the lowest DM loss (*P* < 0.05). Only ATCC14917 treatment enhanced the WSC content (*P* < 0.05). Both bacteriocin-producing strains increased the CP content and decreased the NPN, NH_3_-N, and aNDF contents. All inoculants reduced the ADF content of silage.

**Table 1:** Chemical composition of whole-plant corn silage on d 90

Items^1^	Treatments^2^				SEM^3^	*P*-value
	Control	MTD/1	ATCC14917	CICC24194		
DM, g/kg FW	293^c^	302^b^	305^ab^	307^a^	1.065	< 0.001
DM loss, g/kg DM	78.2^a^	53.7^b^	44.4^c^	39.9^c^	0.351	< 0.001
WSC, g/kg DM	32.7^b^	35.0^b^	45.9^a^	39.4^ab^	1.135	0.007
CP, g/kg DM	50.4^b^	52.6^ab^	55.3^a^	55.1^a^	0.334	0.001
NPN, g/kg TN	647^a^	314^b^	337^b^	305^b^	27.521	0.002
NH_3_-N, g/kg TN	49.6^a^	39.8^ab^	29.6^b^	34.5^b^	1.722	0.009
aNDF, g/kg DM	519^a^	479^ab^	477^b^	476^b^	4.329	0.01
ADF, g/kg DM	326^a^	288^b^	279^b^	283^b^	4.805	0.018
Starch, g/kg DM	209	211	213	217	4.623	0.914

^1^*DM* Dry matter, *FW* Fresh weight, *DM loss* Dry matter loss, *WSC* Water-soluble carbohydrates, *CP* Crude protein, *NPN* Non-protein nitrogen, *TN* Total nitrogen, *NH*_*3*_*-N* Ammonia nitrogen, *aNDF* Neutral detergent fiber with heat-stable α-amylase, *ADF* Acid detergent fiber

^2^Control, distilled water; MTD/1, MTD/1 treatment; ATCC14917, ATCC14917 treatment; CICC24194, CICC24194 treatment

^3^*SEM *Standard error of the means

^a–c^Means within the same row with different superscript letters differ (*P* < 0.05)

### Parameters of bacterial community of whole-plant corn silage inoculated with different LAB strains

Table 2 presents the Shannon index parameters for the WPCS. The interaction between the treatments and ensiling periods affected the Shannon index (*P* < 0.05). With the extension of fermentation time, the Shannon index decreased gradually, regardless of the treatment (*P* < 0.05). The control group had the highest Shannon index throughout the fermentation period, except during the initial ensiling period (d 3) (*P* < 0.05). The lowest Shannon index was observed in the ATCC14917 treatment on d 7 and 90, whereas the Shannon index in the MTD/1 treatment was the lowest on d 60. Compared with the control group, the MTD/1 and ATCC14917 groups decreased the Shannon index values at each ensiling period, except for d 3 and 7.

**Table 2:** The variations of bacterial community alpha-diversity (Shannon index) of whole-plant corn silage

Treatments^1^	Ensiling periods					SEM^2^	Effects^3^		
	3 d	7 d	14 d	60 d	90 d		T	D	T × D
Control	3.89	4.62^a^	4.34^a^	4.23^a^	3.48^a^	0.021	< 0.001	< 0.001	< 0.001
MTD/1	4.16	4.36^ab^	3.93^b^	2.60^c^	2.30^b^				
ATCC14917	4.33	4.23^b^	4.38^a^	3.77^b^	1.86^c^				
CICC24194	4.24	4.46^ab^	4.00^b^	3.70^b^	2.60^b^				
Means	4.15	4.42	4.16	3.53	2.56				

^1^Control, distilled water; MTD/1, MTD/1 treatment; ATCC14917, ATCC14917 treatment; CICC24194, CICC24194 treatment

^2^*SEM *Standard error of the means

^3^T, the effect of treatments; D, the effect of ensiling periods; T × D, the interaction of treatments and ensiling periods

^a–c^Means within the same row with different superscript letters differ (*P* < 0.05)

**Fig. 2:**
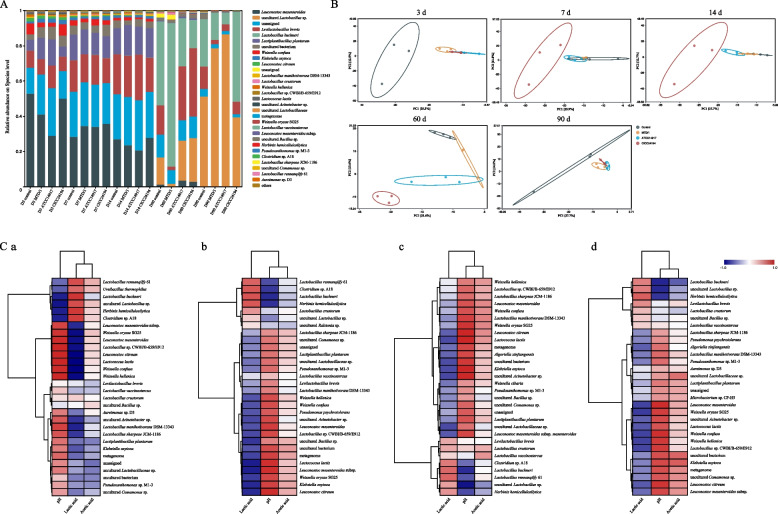
Parameters of (**A**) bacterial community composition, (**B**) principal component analysis (PCA), and (**C**) correlation heatmap between the fermentation characteristics and the top 30 most abundant species of whole-plant corn silage. C: a, control; b, MTD/1 treatment; c, ATCC14917 treatment; d, CICC24194 treatment. Control, distilled water; MTD/1, MTD/1 treatment; ATCC14917, ATCC14917 treatment; CICC24194, CICC24194 treatment

The parameters of the bacterial community composition in WPCS are shown in Fig. 2A. During the first 14 d of ensiling, the dominant species in all groups were *Leuconostoc mesenteroides*, *L. plantarum*, and *Levilactobacillus brevis*. However, when fermentation reached d 60, the dominant species in all groups were uncultured *Lactobacillus* sp., *Lentilactobacillus buchneri*, and *L. brevis*. The relative abundance of *L. mesenteroides* was decreased by the ATCC14917 strain on d 3 and 14 of ensiling, whereas the relative abundance of *L. plantarum* was increased in the MTD/1 and ATCC14917 groups on d 3. On d 60 of silage fermentation, both bacteriocin-producing strains had a lower relative abundance of *L. buchneri* and a higher relative abundance of *L. brevis* compared with that in the control group, whereas the opposite result was observed in the MTD/1 treated silage. In the late ensiling period (d 90), an increased relative abundance of uncultured *Lactobacillus* sp. was observed in the MTD/1 and ATCC14917 treated silages, whereas the relative abundance of *L. buchneri* was increased by the CICC24194 strain.

The results of operational taxonomic unit-based principal component analysis (PCA) are shown in Fig. 2B. At the initial and late ensiling periods (d 3 and 90), the control group was separated from the other groups, whereas at the middle stage of fermentation (7–60 d), the CICC24194 treatment was distinguished from the other groups.

The correlation heatmap between the fermentation characteristics and the top 30 most abundant species in WPCS is shown in Fig. 2C. The relative abundance of *L. buchneri* was negatively correlated with the pH, but positively correlated with the lactic acid concentration in all groups (Fig. 2C); however, a completely inverse outcome was observed for the relative abundances of* L. mesenteroides* and* L. plantarum* in all groups. The lactic acid concentration was positively correlated with the relative abundance of *L. brevis* in both bacteriocin-producing strain-treated silages (Fig. 2C).

**Fig. 3:**
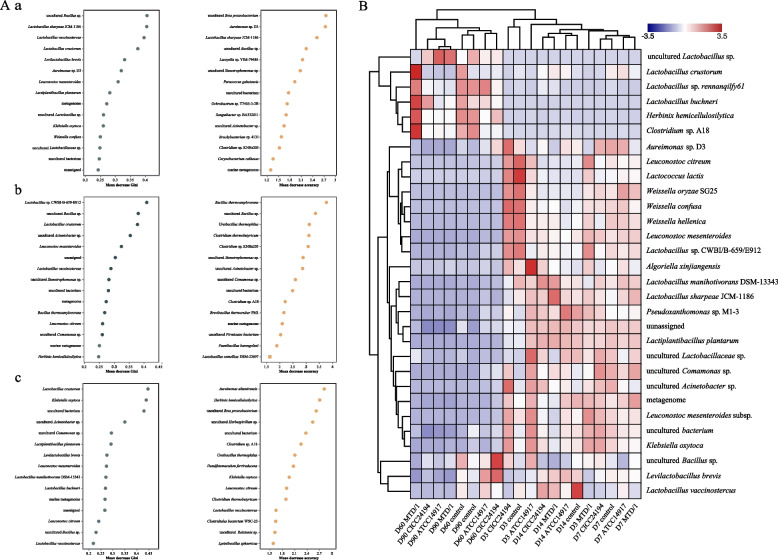
The combined analysis of (**A**) random forest and (**B**) the top 30 abundant species clustering of bacterial communities of whole-plant corn silage. The common top 5 highest-ranking variables of both mean decrease Gini and mean decrease accuracy in random forests were analyzed. A: a, the variations from control to MTD/1 group; b, the variations from control to ATCC14917 group; c, the variations from control to CICC24194 group. Control, distilled water; MTD/1, MTD/1 treatment; ATCC14917, ATCC14917 treatment; CICC24194, CICC24194 treatment

The combined analysis of random forest and the top 30 most abundant species clusters of bacterial communities in WPCS are shown in Fig. 3. In both mean decrease Gini (MDG) and mean decrease accuracy (MDA) cases, uncultured *Bacillus* sp. and *Lacticaseibacillus sharpeae* JCM-1186 were among the top 5 highest-ranking variables in the control to MTD/1 groups (Fig. 3A). Combined with the clustering analysis of species abundance, the relative abundance of uncultured *Bacillus* sp. was decreased by the MTD/1 treatment compared with that in the control group at each fermentation period, except for d 7, while the MTD/1 treated silage increased the relative abundance of *L. sharpeae* JCM-1186 at the early and middle stages of ensiling (d 3 to 14; Fig. 3B). The combined MDG and MDA models showed that uncultured *Bacillus* sp. was the second most influential from the control group to the ATCC14917 treatment (Fig. 3A), while the relative abundance of uncultured *Bacillus* sp. was decreased by the ATCC14917 treatment during ensiling, except for d 3 (Fig. 3B). The MDG revealed that uncultured bacterium was the third most important variable from the control group to the CICC24194 treatment, and for MDA, it was also the fifth most important variable (Fig. 3A), while the CICC24194 treatment decreased the relative abundance of the uncultured bacterium in the late ensiling period (90 d; Fig. 3B).

### In vitro ruminal fermentation characteristics and CH_4_ productions of whole-plant corn silage inoculated with different LAB strains

The in vitro ruminal fermentation parameters are listed in Table 3. The treatment with both bacteriocin-producing strains increased the in vitro DM digestibility (IDVMD), NH_3_-H, propionate, and isovalerate concentrations compared to those in the control group (*P* < 0.05), whereas a decreased total gas production and an increased valerate concentration were observed in the CICC24194 treatment (*P* < 0.05). All 3 silages showed a decreasing trend in the CH_4_ production, CH_4_ to total gas ratio, acetate to propionate, acetate, and butyrate concentrations (*P* < 0.05), whereas the CH_4_ production, CH_4_ to total gas ratio, and butyrate concentrations in both bacteriocin-producing strain-treated silages were lower than those in the MTD/1 treated silage. The MTD/1 and ATCC14917 treatments decreased the total volatile fatty acid concentration.

**Table 3:** In vitro rumen fermentation parameters and methane concentration of whole-plant corn silage

Items^1^	Treatments^2^				SEM	*P*-value
	Control	MTD/1	ATCC14917	CICC24194		
pH	6.65	6.58	6.51	6.50	0.020	0.093
NH_3_-H, mg/100 mL	19.4^b^	21.5^b^	25.6^a^	27.3^a^	0.449	0.001
IDVMD, g/kg DM	51.9^c^	53.3^bc^	55.0^ab^	55.8^a^	0.237	0.002
Total gas, mL/g	124^a^	116^ab^	110^ab^	109^b^	1.590	0.040
CH_4_, mL/g	12.8^a^	10.7^b^	9.15^c^	8.95^c^	0.129	< 0.001
CH_4_-to-total gas, % mL	10.3^a^	9.15^b^	8.28^c^	8.18^c^	0.051	< 0.001
Total VFA, mmol/L	49.8^a^	47.6^b^	47.8^b^	48.7^ab^	0.176	0.008
Acetate, mmol/L	31.9^a^	29.8^b^	29.5^b^	28.0^c^	0.139	< 0.001
Propionate, mmol/L	6.35^c^	7.37^bc^	8.5^ab^	10.0^a^	0.231	0.003
Acetate to propionate	5.05^a^	4.06^b^	3.48^bc^	2.81^c^	0.108	< 0.001
Butyrate, mmol/L	8.80^a^	7.88^b^	6.80^c^	6.45^c^	0.102	< 0.001
Isobutyrate, mmol/L	0.26	0.23	0.27	0.25	0.007	0.378
Valerate, mmol/L	0.97^b^	0.85^b^	0.88^b^	1.42^a^	0.020	< 0.001
Isovalerate, mmol/L	1.48^c^	1.39^c^	1.90^b^	2.47^a^	0.039	< 0.001

^1^*NH*_*3*_*-H* Ammonia, *DM *Dry matter, *IDVMD* In vitro DM digestibility, *CH*_*4*_ Methane, *VFA* Volatile fatty acids

^2^Control, distilled water; MTD/1, MTD/1 treatment; ATCC14917, ATCC14917 treatment; CICC24194, CICC24194 treatment

^3^*SEM* Standard error of the means

^a–c^Means within the same row with different superscript letters differ (*P* < 0.05)

### In vitro ruminal bacterial communities of whole-plant corn silage inoculated with different LAB strains

**Fig. 4:**
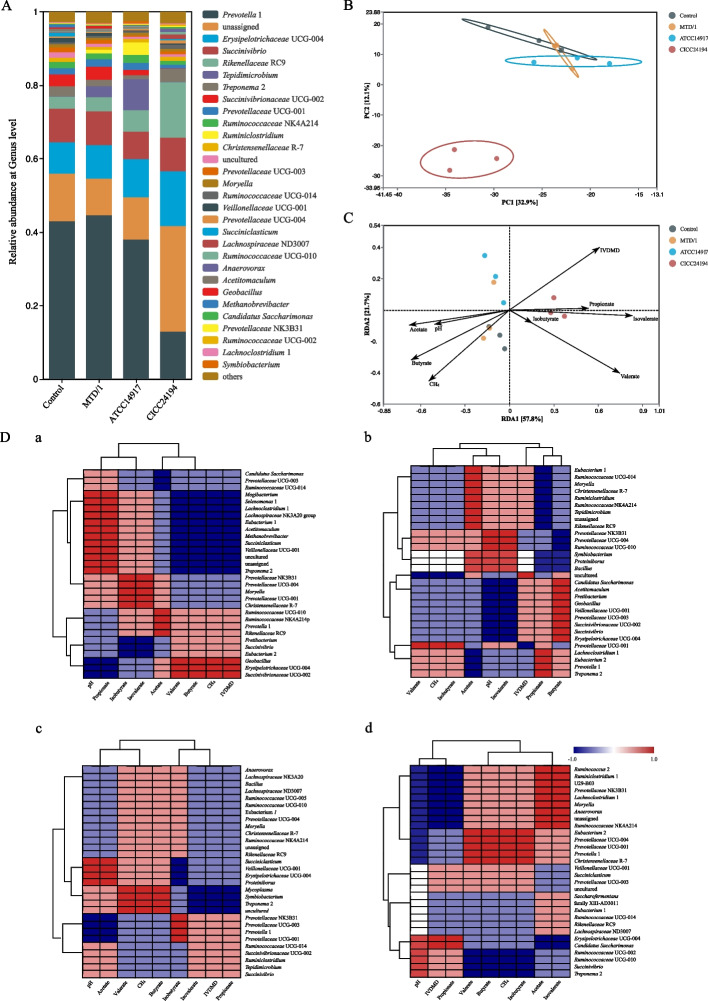
In vitro rumen (**A**) bacterial community composition, (**B**) principal component analysis (PCA), (**C**) redundancy analysis (RDA), and (**D**) correlation heatmap between the fermentation characteristics and the top 30 most abundant genera of whole-plant corn silage. The length of the arrow of RDA represents the degree of influence on the treatments; the angle between the arrows represents positive and negative correlation (acute angle: positive correlation; obtuse angle: negative correlation; right angle: no correlation); the distance between the projected point and the origin represents the influence of the fermentation characteristics on the distribution of the treatments. D: a, control; b, MTD/1 treatment; c, ATCC14917 treatment; d, CICC24194 treatment. Control, distilled water; MTD/1, MTD/1 treatment; ATCC14917, ATCC14917 treatment; CICC24194, CICC24194 treatment

The composition of the bacterial community during in vitro ruminal fermentation is shown in Fig. 4A. The MTD/1 and ATCC14917 treatments increased the relative abundances of *Tepidimicrobium* and *Ruminiclostridium*, whereas the relative abundances of the two genera in the ATCC14917 treatment were the highest. The relative abundance of *Prevotella* 1 decreased in the CICC24194 treated silage compared with that in the other groups, whereas an increased relative abundance of *Rikenellaceae* RC9 and *Treponema* 2 was observed in the CICC24194 treatment. Both bacteriocin-producing strains decreased the relative abundance of *Succinivibrionaceae* UCG-002 compared with that in the other groups, and the relative abundance of *Succinivibrionaceae* UCG-002 was the lowest in the CICC2414 treatment. Additionally, the ruminal bacterial community in the CICC24194 group was distinctly separated from that in the other groups (Fig. 4B).

The redundancy analysis (RDA) between rumen fermentation parameters and treatments is shown in Fig. 4C. The CICC24194 treatment was positively correlated with the IVDMD, propionate, isobutyrate, valerate, and isovalerate concentrations, whereas the control and MTD/1 groups were positively correlated with the pH, acetate, butyrate, and CH_4_ concentrations.

The correlation heatmap between the in vitro ruminal fermentation characteristics and the top 30 most abundant genera is shown in Fig. 4D. The relative abundances of *Tepidimicrobium* and *Ruminiclostridium* in the MTD/1 treatment were positively correlated with the IVDMD and acetate concentration, and negatively correlated with the CH_4_ and propionate concentrations, whereas the two genera in the ATCC14917 treatment were positively correlated with the IVDMD, acetate, and propionate concentrations, and negatively correlated with the CH_4_ production. The relative abundance of *Rikenellaceae* RC9 in the CICC24194 treated silage was positively correlated with the acetate and isovalerate concentrations, and negatively correlated with the IVDMD, CH_4_, and propionate concentrations. Moreover, the relative abundance of *Treponema* 2 in the CICC24194 treatment was positively correlated with the IVDMD and propionate concentration, and negatively correlated with the CH_4_ and acetate concentrations.

**Fig. 5:**
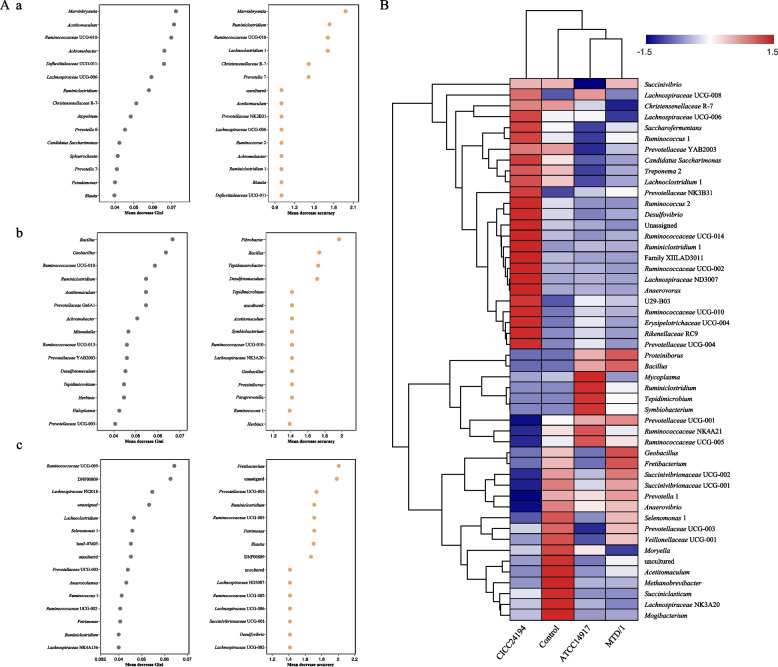
The combined analysis of (**A**) random forest and (**B**) the top 30 most abundant genera clustering of bacterial communities of in vitro rumen fermentation of whole-plant corn silage. The common top 5 highest-ranking variables of both mean decrease Gini and mean decrease accuracy in random forests were analyzed. A: the variations from a, control to MTD/1 group; b, the variations from control to ATCC14917 group; c, the variations from control to CICC24194 group. Control, distilled water; MTD/1, MTD/1 treatment; ATCC14917, ATCC14917 treatment; CICC24194, CICC24194 treatment

The combined analysis of the random forest and the top 30 most abundant genera clustering of bacterial communities during in vitro rumen fermentation are shown in Fig. 5. *Marvinbryantia* and *Ruminococcaceae* UCG-010 were among the top one and the top 3 highest-ranking variables in both MDG and MDA from the control group to the MTD/1 treatment (Fig. 5A). According to the clustering analysis of genus abundance (Fig. 5B), the relative abundance of *Ruminococcaceae* UCG-010 in the MTD/1 treatment was higher than that in the control group, whereas the abundance of *Marvinbryantia* was lower in the MTD/1 treated silage compared with that in the control group (Fig. S1). *Bacillus* was the first and second most influential genus in MDG and MDA from the control group to the ATCC14917 treatment, respectively (Fig. 4A), while its relative abundance increased in the ATCC14917 treated silage compared with that in the control group (Fig. 5B). *Ruminococcaceae* UCG-005 was the first and fifth variable in MDG and MDA from the control group to the CICC24194 treatment, respectively (Fig. 5A). Combined with the cluster analysis of genus abundance, a decreased relative abundance of *Ruminococcaceae* UCG-005 was observed in the CICC24194 treatment group (Fig. 5B).

### In vitro ruminal microbial populations of whole-plant corn silage inoculated with different LAB strains

The populations of total bacteria, methanogens, protozoa, and fungi are presented in Table 4. Both bacteriocin-producing strains decreased the populations of the total bacteria and fungi compared with those in the control group and the MTD/1 treatment (*P* < 0.05), whereas the lowest total bacterial and fungal populations were observed in the CICC24194 treatment (*P* < 0.05). Only CICC24194 treated silage reduced the methanogen population (*P* < 0.05). The protozoan population decreased in all 3 strain-treated silages (*P* < 0.05), with the CICC24194 treatment having the lowest protozoan population (*P* < 0.05).

**Table 4:** In vitro rumen microbial populations of whole-plant corn silage

Items	Treatments^1^				SEM^2^	*P*-value
	Control	MTD/1	ATCC14917	CICC24194		
Total bacteria, log_10_ copies/mL	8.93^a^	8.88^a^	8.74^b^	8.41^c^	0.010	< 0.001
Fungi, log_10_ copies/mL	6.16^a^	5.77^b^	5.29^c^	4.99^d^	0.026	< 0.001
Methanogens, log_10_ copies/mL	8.12^a^	7.98^a^	7.98^a^	7.43^b^	0.041	0.001
Protozoa, log_10_ copies/mL	6.22^a^	5.73^b^	5.35^bc^	5.16^c^	0.042	< 0.001

^1^Control, distilled water; MTD/1, MTD/1 treatment; ATCC14917, ATCC14917 treatment; CICC24194, CICC24194 treatment

^2^*SEM* Standard error of the means

^a−d^Means within the same row with different superscript letters differ (*P* < 0.05)

## Discussion

### Effect of bacteriocin-producing strains on fermentation profile, chemical composition, and bacterial community of whole-plant corn silage

In comparison with control or MTD/1 treatments, the higher pH in the bacteriocin-producing strains inoculated silage fermented for 3 d suggested that bacteriocin-producing strains could inhibit silage fermentation at initial fermentation stage; however, the increased lactic acid in the bacteriocin-producing strains inoculated silage fermented for 7 d indicated that silage fermentation was promoted by both bacteriocin-producing strains after 3 d of fermentation. Butyric acid was completely absent in all groups, while propionic acid was only present in the control group, suggesting the 3 inoculants improved the silage fermentation quality compared to the control group. In addition, the highest DM content in the CICC24194 treatment and the lowest DM loss in both bacteriocin-producing strains treatments suggested that the bacteriocins produced by the 2 strains effectively suppressed the growth of other microbes that may consume nutrients, as evidenced by the minimal variation in pH among the groups. These results was consistent with the previous studies [11]. Moreover, the ATCC14917 treatment had a higher WSC content, but similar aNDF and ADF contents compared with those in the MTD/1 treatment, which also could be attributed to the bacteriocin-producing ability of the ATCC14917 strain [27]. A similar effect was observed for the N fraction of the silages. The increased CP content and the decreased NPN and NH_3_-N contents in the ATCC14917 and CICC24194 treatments consistent with the results that the 2 bacteriocin-producing strains minimized silage nutrients loss and proteolysis during ensiling [11, 12].

Regardless of the treatment, the decrease in Shannon index with advancing fermentation period indicated a gradual decrease in bacterial diversity [28]. The lowest Shannon index value corresponded to the lowest pH in the MTD/1 treatment from d 14 to 60 of ensiling. However, the opposite results were observed for the CICC24194 treatment on d 14 and the ATCC14917 treatment on d 90, which had the highest pH and lowest Shannon index values. In summary, the antibacterial effects of bacteriocins produced by the 2 strains reduced the bacterial diversity [12]. Moreover, the separation of the CICC24194 treated group from the other groups in PCA implied its antibacterial capacity.

The dominant bacterial species, *L. mesenteroides*, *L. plantarum*, and *L. brevis* in all groups before 14 d were replaced by uncultured *Lactobacillus* sp., *L. buchneri*, and *L. brevis* after 14 d, which was consistent with a previous study [29]. This indicated that the tolerance of *L. buchneri* to acidic environments played an influential role in the late stages of fermentation. Further, inoculation with the 2 bacteriocin-producing strains preserved *L. brevis* fermentation on d 60 of ensiling, corresponding to the high lactic acid production during ensiling in the CICC24194 treatment [30]. This result was confirmed by the correlation heatmap between the fermentation characteristics and species of WPCS, where the relative abundance of *L. brevis* was positively correlated with the lactic acid production in the silages treated with both bacteriocin-producing strains, indicating that the 2 bacteriocin-producing strains facilitated the fermentation by *L. brevis.* In addition, the relative abundance of *L. buchneri* increased in the MTD/1 treated silage on d 60 and the control and MTD/1 groups on d 90. This result was consistent with the highest acetic acid content observed in the MTD/1 treatment group on d 60 and the control and MTD/1 groups on d 90.

Combined with the clustering of bacterial community analysis, the 5 most common highest-ranking variables in both MDA and MDG from the control group to each treatment in the present study were calculated to analyze the effects of different treatments on influential variables. The relative abundance of the influential ranked variable, uncultured *Bacillus* sp., from the control group to MTD/1 and ATCC14917 treatments in both MDG and MDA decreased in the 2 strain-treated silages. *Bacillus* is one of the main contributors to the reduction of silage quality [31]. This result suggested that the application of MTD/1 and ATCC14917 improved the fermentation quality of WPCS by inhibiting the consumption of nutrients by *Bacillus*. Moreover, the relative abundance of the key variable, uncultured bacterium, from the control group to the CICC24194 treatment was decreased by the CICC24194 strain, indicating a bacteriostatic effect of the strain.

### Effect of bacteriocin-producing strains on in vitro ruminal parameters and microbial community of whole-plant corn silage

As expected, all 3 strain-treated silages showed a reduction in CH_4_ production and acetate concentration, whereas the 2 bacteriocin-producing strains were more effective than MTD/1 strain. This result was attributed to the antibacterial effect of the bacteriocin produced by the 2 strains, which was demonstrated by the decreased Shannon index values in both bacteriocin-producing strain-treated silages compared to the MTD/1 treated silage (Table S2). Moreover, a similar outcome was observed in PCA. The CICC24194 treatment was significantly distinguished from the other groups, indicating the variations in bacterial communities in this treatment. The increased IVDMD in both bacteriocin-producing strains was consistent with the increased DM content and decreased DM loss in the 2 silages [32]. In addition, this result was also due to the increase in propionate concentration because energy is efficiently utilized when propionate is produced during rumen fermentation compared to the production of acetate and butyrate, which facilitates CH_4_ production [33]. Similarly, the RDA results showed that the CICC24194 treatment positively correlated with the IVDMD and propionate concentrations and negatively correlated with the CH_4_ production. In addition, according to the correlation heatmap between the in vitro ruminal fermentation properties and the relative abundance of bacterial communities, the increased relative abundance of *Treponema* 2 in the CICC24194 treatment group was positively correlated with the IVDMD and propionate concentrations, and negatively correlated with the CH_4_ production and acetate concentration. The main fermentative end-product of *Treponema* is succinate, whose metabolic pathway tends to produce propionate instead of CH_4_ [33, 34]. The increase in IVDMD and propionate concentration in the ATCC14917 treatment may be due to its positive correlation with the higher relative abundance of *Tepidimicrobium*, which had the ability to convert glucose into propionate. Interestingly, the metabolites of *Tepidimicrobium* included acetate, and the acetate concentration decreased in the ATCC14917 treatment. Therefore, the abundance values of genera that showed an obvious positive or negative correlation with the acetate concentration in the ATCC14917 treatment were analyzed in this study (Table S3). The abundance values of *Succiniclasticum* and *Erysipelotrichaceae* UCG-004 in the ATCC14917 treatment were the lowest and positively correlated with the acetate concentrations. This result suggested that the decreased acetate concentration in the ATCC14917 treatment was attributed to the decreased abundance of *Succiniclasticum* and *Erysipelotrichaceae* UCG-004.

The relative abundance of the first variable *Marvinbryantia* decreased in the MTD/1 group compared to that in the control group, which was consistent with the reduction in butyrate concentration in the MTD/1 treatment. This may be due to the fact that *Marvinbryantia* is capable of producing butyrate during fiber metabolism [35]. The relative abundance of the third variable *Ruminococcaceae* UCG-010 decreased in the MTD/1 treated silage compared with that in the control group, which was consistent with the decreased CH_4_ and acetate concentrations in this group [36]. The first and second most influential *Bacillus* in MDA and MDG were increased by the ATCC14917 treatment compared with those in the control group, which was consistent with the increased IDVMD in the ATCC14917 treatment [37]. Similar to the results of the MTD/1 treatment, the CICC24194 treated silage decreased the relative abundance of *Ruminococcaceae* UCG-005 compared with that in the control group, corresponding to the decreased CH_4_ and acetate concentrations.

In addition to methanogens producing CH_4_ directly, protozoa and fungi can provide methanogens with large amounts of H_2_ to produce CH_4_ [38, 39]. Therefore, the highest propionate concentration and the lowest populations of total bacteria, fungi, methanogens, and protozoa in the CICC24194 treatment clarified the lowest CH_4_ concentration in this treatment. This result can be attributed to the antibacterial activity of the CICC24194 strain.

## Conclusion

Both class IIa bacteriocin-producing *L. plantarum* strains improved the fermentation quality of the whole-plant corn silage by regulating the bacterial community composition during ensiling, with CICC24194 being the most effective. In addition, both bacteriocin-producing strains reduced the CH_4_ emissions more effectively than MTD/1, with CICC24144 showing the greatest potential for improving in vitro rumen digestibility. Both bacteriocin-producing strains mitigated the CH_4_ production and improved digestibility by modulating the total bacteria, fungi, methanogens, and protozoan populations, and the composition of fibrolytic bacteria in the rumen.

### Supplementary Information

**Additional file 1:** **Table S1**. Quantitative Real-Time PCR primer information.

**Additional file 2:** **Table S2**. In vitro rumen bacterial Shannon index of whole-plant corn silage.

**Additional file 3:** **Table S3**. The abundance values of in vitro ruminal bacteria at genus level of whole-plant corn silage.

**Additional file 4:** **Fig.** **S1**. Abundance of Marvinbryantia of in vitro rumen fermentation of whole-plant corn silage.

## Data Availability

The datasets used and/or analysed during the current study are available from the corresponding author on reasonable request.

## Abbreviations

AA: Acetic acid
ADF: Acid detergent fiber
CH_4_: Methane
CP: Crude protein
DM: Dry matter
FW: Fresh weight
IDVMD: In vitro DM digestibility
LA: Lactic acid
aNDF: Neutral detergent fiber with heat-stable α-amylase
MDA: Mean decrease accuracy
MDG: Mean decrease Gini
NH_3_-N: Ammonia nitrogen
NPN: Non-protein nitrogen
PA: Propionic acid
PCA: Principal component analysis
RDA: The redundancy analysis
TMR: Total mixed ration
TN: Total nitrogen
VFA: Volatile fatty acids
WPCS: Whole-plant corn silage
WSC: Water-soluble carbohydrates
