# Supplementary material for: Effects of bacteriocin-producing Lactiplantibacillus plantarum on bacterial community and fermentation profile of whole-plant corn silage and its in vitro ruminal fermentation, microbiota, and CH4 emissions
Source: J Anim Sci Biotechnol. 2024 Aug 7;15:107. doi: 10.1186/s40104-024-01065-w (PMC11304621; doi:10.1186/s40104-024-01065-w)
Supplement: Supplementary file 1 — Additional file 1: Table S1. Quantitative Real-Time PCR primer information. [file 40104_2024_1065_MOESM1_ESM.docx]

| **Gene items** | **Primers** | **Primer sequence (5'→3')** | **Length, bp** |
| --- | --- | --- | --- |
| V3V4 | 338F | ACTCCTACGGGAGGCAGCA | 446 |
|  | 806R | GGACTACHVGGGTWTCTAAT | 446 |
| ITS2-1 | ITS3-F | GCATCGATGAAGAACGCAGC | 402 |
|  | ITS4-R | TCCTCCGCTTATTGATATGC | 402 |
| mcrA-1 | MLf | GGTGGTGTMGGATTCACACARTAYGCWACAGC | 472 |
|  | MLr | TTCATTGCRTAGTTWGGRTAGTT | 472 |
| 18SV4-1 | CHEND-18S-NS1 | ATTCCCCGTTACCCGTTG | 349 |
|  | CHEND-18S-fung | GTAGTCATATGCTTGTCTC | 349 |

**Table S1** Quantitative Real-Time PCR primer information
