# Supplementary material for: Effects of bacteriocin-producing Lactiplantibacillus plantarum on bacterial community and fermentation profile of whole-plant corn silage and its in vitro ruminal fermentation, microbiota, and CH4 emissions
Source: J Anim Sci Biotechnol. 2024 Aug 7;15:107. doi: 10.1186/s40104-024-01065-w (PMC11304621; doi:10.1186/s40104-024-01065-w)
Supplement: Supplementary file 2 — Additional file 2: Table S2. In vitro rumen bacterial Shannon index of whole-plant corn silage. [file 40104_2024_1065_MOESM2_ESM.docx]

| **Treatments^1^** | **Shannon index** | **SEM^2^** | ***P*-value** |
| --- | --- | --- | --- |
| Control | 6.22^a^ | 0.020 | <0.001 |
| MTD/1 | 5.94^b^ |  |  |
| ATCC14917 | 5.68^c^ |  |  |
| CICC24194 | 5.74^c^ |  |  |

**Table S2** In vitro rumen bacterial Shannon index of whole-plant corn silage

^1^Control, distilled water; MTD/1, MTD/1 treatment; ATCC14917, ATCC14917 treatment; CICC24194, CICC24194 treatment

^2^*SEM* Standard error of the means

^a−c^Means within the same row with different superscript letters differ (*P* < 0.05)
