# Supplementary material for: Effects of bacteriocin-producing Lactiplantibacillus plantarum on bacterial community and fermentation profile of whole-plant corn silage and its in vitro ruminal fermentation, microbiota, and CH4 emissions
Source: J Anim Sci Biotechnol. 2024 Aug 7;15:107. doi: 10.1186/s40104-024-01065-w (PMC11304621; doi:10.1186/s40104-024-01065-w)
Supplement: Supplementary file 3 — Additional file 3: Table S3. The abundance values of in vitro ruminal bacteria at genus level of whole-plant corn silage. [file 40104_2024_1065_MOESM3_ESM.docx]

**Table S3** The abundance values of in vitro ruminal bacteria at genus level of whole-plant corn silage

| **Items** | **Treatments**^1^ | | | | **SEM**^2^ | ***P*-values** |
| --- | --- | --- | --- | --- | --- | --- |
|  | **Control** | **MTD/1** | **ATCC14917** | **CICC24194** |  |  |
| *Prevotellaceae* NK3B31 | 157 | 144 | 173 | 172 | 6.823 | 0.446 |
| *Prevotellaceae* UCG-003 | 901^a^ | 526^b^ | 274^c^ | 518b^c^ | 27.375 | < 0.001 |
| *Prevotella* 1 | 35,655^a^ | 36,624^a^ | 29,883^a^ | 9,965^b^ | 1,025.516 | < 0.001 |
| *Prevotellaceae* UCG-001 | 1,387^a^ | 1,655^a^ | 1,505^a^ | 793^b^ | 50.6 | 0.002 |
| *Succiniclasticum* | 1,368^a^ | 258^b^ | 67.31^c^ | 301^b^ | 16.064 | < 0.001 |
| *Veillonellaceae* UCG-001 | 1,238^a^ | 902^a^ | 90.5^b^ | 266^b^ | 70.0 | 0.001 |
| *Erysipelotrichaceae* UCG-004 | 9,419^ab^ | 9,237^ab^ | 7,841^c^ | 13,371^a^ | 511 | 0.025 |
| *Proteiniborus* | 1.67 | 1.33 | 0.67 | 0.67 | 0.17 | 0.16 |

^1^Control, distilled water; MTD/1, MTD/1 treatment; ATCC14917, ATCC14917 treatment; CICC24194, CICC24194 treatment

^2^*SEM* Standard error of the means

^a−c^Means within the same row with different superscript letters differ (*P* < 0.05)
