# Supplementary figures and images for: Effects of bacteriocin-producing Lactiplantibacillus plantarum on bacterial community and fermentation profile of whole-plant corn silage and its in vitro ruminal fermentation, microbiota, and CH4 emissions
Source: J Anim Sci Biotechnol. 2024 Aug 7;15:107. doi: 10.1186/s40104-024-01065-w (PMC11304621; doi:10.1186/s40104-024-01065-w)

# *Marvinbryantia*

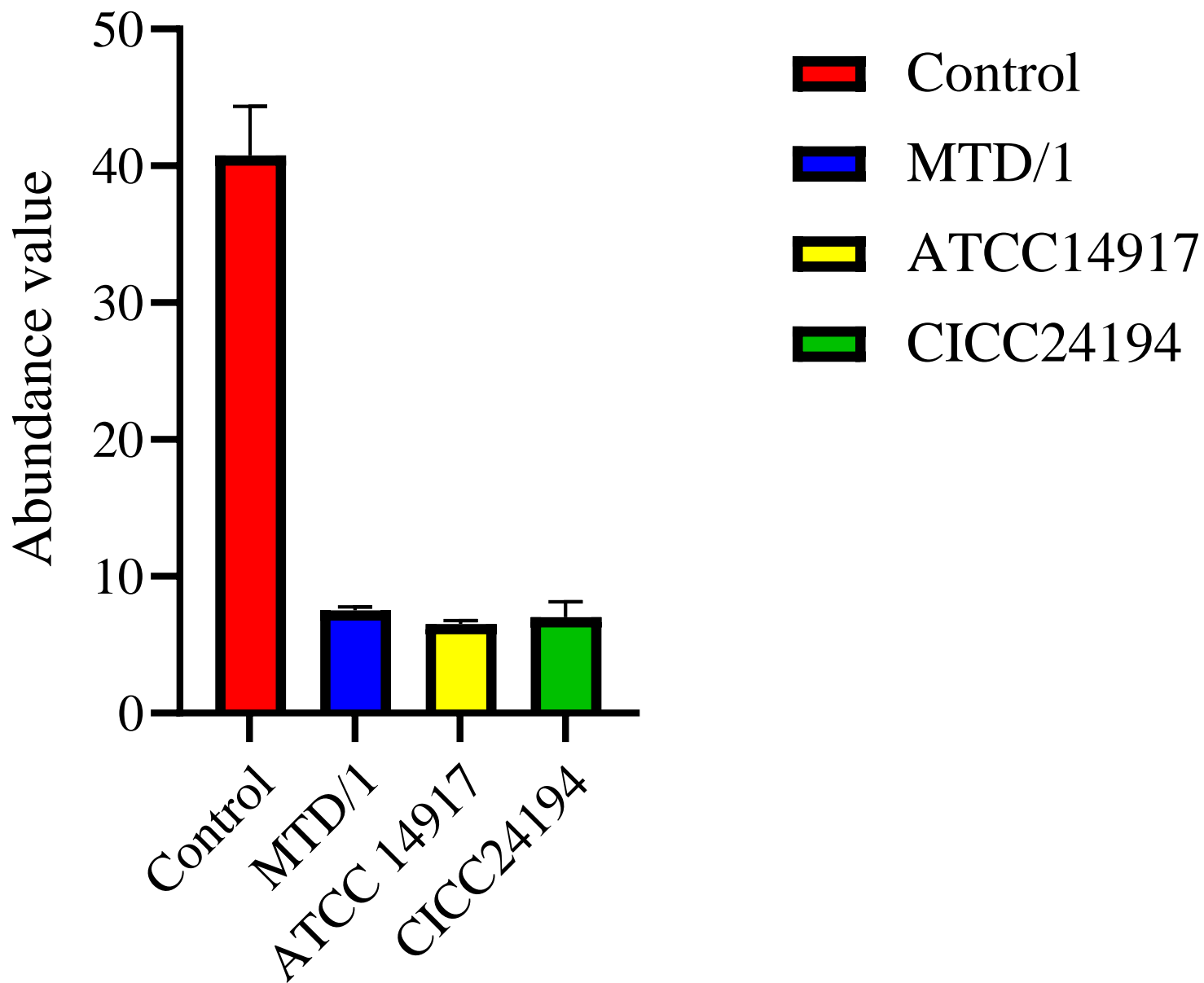

Supplement: Supplementary file 4 — Additional file 4: Fig. S1. Abundance of Marvinbryantia of in vitro rumen fermentation of whole-plant corn silage. [file 40104_2024_1065_MOESM4_ESM.pdf]
